# Needs, Challenges, and Opportunities for Genetic Counselors in Vietnam: A Qualitative Interview Study With Healthcare Leaders and Genetics Professionals

**DOI:** 10.1002/jgc4.70303

**Published:** 2026-10-10

**Authors:** Mai Ly Tran Burke, Peter James B. Abad, Basma M'Barek, Angelica Michelle Raney, Janine S. Bruce, Sylvia Bereknyei Merrell, Hetanshi Naik, Kelly E. Ormond

**Affiliations:** ^1^ Department of Genetics Stanford University School of Medicine Stanford California USA; ^2^ College of Nursing University of the Philippines Manila Manila Philippines; ^3^ FV Hospital Ho Chi Minh City Vietnam; ^4^ Department of Pediatrics Stanford University School of Medicine Stanford California USA; ^5^ Department of Health Sciences and Technology, Health Ethics and Policy Lab ETH Zürich Zürich Switzerland

## Abstract

In Vietnam, access to genetic testing has expanded rapidly, but the genetic counseling needed to translate genetic testing results into informed healthcare decisions remains limited. Currently, genetic counseling is provided by physicians or PhD‐trained laboratory geneticists in Vietnam; there are no Master's‐level trained genetic counselors or genetic counseling training programs. As such, significant gaps exist in the genetic counseling services offered to patients and families in Vietnam. To date, limited research has explored how to implement the profession of genetic counselors in Vietnam. This study, therefore, explores the needs, challenges, and opportunities for integrating the profession of genetic counselors into Vietnam's healthcare system from the perspectives of healthcare professionals, institutional leaders, and genetics specialists. Grounded in a constructivist paradigm and the Consolidated Framework for Implementation Research (CFIR), we conducted 25 semi‐structured interviews with healthcare professionals, institutional leaders, and genetics specialists across Northern, Central, and Southern Vietnam in 2025. We purposively sampled participants to represent diverse roles, clinical populations, and institutions, including public and private hospitals, testing laboratories, advocacy organizations, and public health agencies. We collected demographic data via a screening survey and conducted virtual interviews in Vietnamese or English. Using inductive, reflexive thematic analysis, we analyzed transcripts and mapped themes to CFIR to support actionability. Participants described three major themes: (1) genetic testing is outpacing genetics specialists; (2) family, spiritual, and hierarchical frameworks shape the patient response to genetic care; and (3) a phased implementation pathway for genetic counselors is needed. Participants converged on a roadmap that includes raising awareness, embedding genetic counselors within existing clinical pathways, developing short‐ and long‐term training strategies, and securing Ministry of Health recognition and guidelines. These findings constitute the first locally grounded implementation roadmap for genetic counselors in Vietnam.

## Plain Language Summary

We interviewed 25 healthcare providers and leaders across Vietnam to understand where genetic counselors are needed most and how the profession of genetic counselors can be sustainably introduced. Participants agreed that genetic counselors can help many families, as genetic results are currently not leading to prevention or action, and counseling is culturally and socially complex. But if genetic counselors are to benefit patients in Vietnam, training, guidelines, and awareness are critical.

**What Is Known About This Topic**

Genetic testing is rapidly expanding in Vietnam, but there are not yet any formally trained genetic counselors or genetic counseling training programs. This has left many gaps in the provision of genetic counseling services across prenatal, cancer, and medical genetics care.

**What This Paper Adds to the Topic**

This study provides the first needs assessment for the profession of genetic counselors in Vietnam. Supported by an implementation framework and a diversity of key healthcare leaders and genetics professionals from across the country, it highlights both the need for genetic counselors and actionable steps to establish the field sustainably.

## 1 Introduction

The global landscape of genetic medicine has advanced rapidly. The cost of sequencing a single human genome has fallen from nearly 100 million dollars in 2001 to several hundred dollars today, making genetic testing more accessible to clinicians around the world (Karn 2013; Illumina Inc. 2023). This technology has transformed the ability to predict, prevent, and personalize treatment for a vast range of conditions (Khan et al. 2025). Yet the benefits of genetic testing can only be fully realized when patients have access to professionals to help them understand, interpret, and act on their results. The National Society of Genetic Counselors (NSGC) defines genetic counseling as “the process of helping people understand and adapt to the medical, psychological, and familial implications of genetic contributions to disease” (Resta et al. 2006, 77). The World Health Organization (WHO) emphasizes that genetic counseling is an “indivisible part of the delivery of genetic services” and recommends its integration into public health systems in developing nations (Ballantyne et al. 2006, 22). Genetic counseling has led to measurable public health benefits across countries in Asia—in Singapore, for example, genetic counseling through a voluntary prenatal screening program supported informed reproductive decision‐making and was associated with a 15‐ to 20‐fold decrease in severe thalassemia births (Tan 2005; Yeo 2004). In the Philippines, telegenetic counseling through the national newborn screening network improved access to genetic services for hundreds of families with genetic conditions in remote areas, enabling guidance on recurrence risks and management (Padilla et al. 2024). However, in many low‐ and middle‐income countries (LMICs), including Vietnam, the profession of genetic counselors remains underdeveloped (Thong et al. 2018), underrecognized (Lim et al. 2025), and inaccessible to the general population (Laurino et al. 2018).

Vietnam presents a compelling case for a needs assessment to inform the development of the genetic counselor profession. Located in Southeast Asia, it operates a mixed public‐private healthcare system organized into tiered service delivery–central hospitals, provincial and district clinics, commune health stations, and village health workers–through which genetic services can access patients across the country (Ormiston and Hanson 2023). Its rapid economic growth has ushered in a new era of genetic testing, with growing investments in genomic technologies and the installation of 66 gene sequencers nationwide by 2022 (Ormiston and Hanson 2023). Yet the human capacity to deliver genetic counseling has lagged behind: applying the Royal College of Physicians' recommendation of 6–12 genetic counselors per 1 million people, Vietnam would require 600–1200 genetic counselors to adequately serve its population (Cutiongco‐de la Paz et al. 2019). However, in 2018, only 13 geneticists and no formally trained genetic counselors served a population of over 90 million (Leppig 2018).

Prior studies have highlighted areas where genetic counselors might address unmet needs in medical genetics, prenatal, and cancer care in Vietnam. For example, in medical genetics, Vietnam has a heavy burden of rare, heritable conditions; its population carries among the highest global rates for some recessive conditions, including the thalassemias, with some estimates approaching 50% carriers (Tran et al. 2021; Goh et al. 2020; Bach et al. 2022). X‐linked and autosomal recessive disorders like these contribute to almost a quarter of total neonatal mortality in Vietnam (To‐Mai et al. 2024), and individuals with these conditions often require intensive lifelong care. Vietnam does not yet have an official Vietnamese‐specific carrier screening panel (To‐Mai et al. 2024). Vietnam does, however, operate a national newborn screening program available across public hospitals, with a government target to screen 90% of newborns by 2030 (Therrell et al. 2025). Effective July 2026, national guidance recommends screening for at least 29 genetic conditions, including spinal muscular atrophy, severe combined immunodeficiency, and thalassemia (Ministry of Health of Vietnam 2026). Positive screens require counseling and follow‐up, yet published descriptions do not indicate who provides it, particularly given the absence of formally trained genetic counselors in Vietnam. In prenatal care, although congenital defects remain the leading cause of infant mortality nationally (Canh Chuong et al. 2018), one hospital‐based study found that most pregnant women who underwent prenatal diagnostic testing did not receive counseling on the test or its implications (Ha et al. 2017). Beyond inherited conditions, genetic counselors are trained to counsel on medication, infectious, and environmental contributors to congenital anomalies (Accreditation Council for Genetic Counseling 2023): in Vietnam, folic acid is typically recommended only from 16 weeks, well past the window in which it can prevent neural tube defects in fetuses (Ha et al. 2019). In cancer care, cascade testing is often missed: among patients in Vietnam who tested positive for high‐risk hereditary cancer syndromes, fewer than half had family members pursue cascade genetic testing (Nguyen et al. 2023). Genetic counselors are uniquely positioned to address these critical gaps through informative patient counseling, coordinating cascade testing for at risk families, and developing population‐specific carrier screening programs (Sagaser et al. 2023; Jacobs et al. 2024).

Although some clinical genetic services are available at hospitals like the National Hospital of Pediatrics in Hanoi and Hue University of Medicine and Pharmacy, genetic counseling is typically provided by PhD‐trained geneticists or physicians in laboratory or clinical settings, rather than by specifically trained genetic counselors (Laurino et al. 2017; Leppig 2018). Some medical schools in Vietnam offer limited, foundational genetics education, offering approximately 45 h of medical genetics lectures for medical students and 40 h for postgraduates (Laurino et al. 2017). More specialized genetics education opportunities are emerging, such as biomedical genetics residency training (Phòng Quản lý Đào tạo Sau đại học, Trường Đại học Y Hà Nội 2023) and continuing education in cancer genetic counseling for physicians, covering foundational topics such as hereditary cancer risk assessment, pedigrees, and targeted therapies for common cancers (Trung tâm Y Sinh học Phân tử 2025). However, no Master's training program for genetic counselors—the global standard for training (Ormond et al. 2024)–currently exists in the country as of 2026.

To date, there has been no research exploring how the profession of genetic counselors can be successfully integrated into Vietnam's healthcare system. This study seeks to fill this gap by addressing the following primary research question: What are the needs, challenges, and opportunities for establishing the profession of genetic counselors within Vietnam's healthcare system, as perceived by healthcare leaders and genetics professionals in 2025? Sub‐questions include (1) How do current healthcare professionals in Vietnam perceive the role and value of genetic counselors in clinical care? and (2) What systemic, cultural, logistical, and infrastructural factors act as barriers or facilitators to the development of the genetic counselor profession in Vietnam?

As the first needs assessment of its kind in Vietnam, this project aims to lay the groundwork for developing training programs and credentialing pathways, regulatory frameworks and practice standards, and clinical integration models that will help bring the profession of genetic counselors to life in Vietnam.

## 2 Methods

### 2.1 Design

Using a descriptive qualitative design (O'Brien et al. 2014) and guided by a constructivist paradigm, we explored the perceived needs, challenges, and opportunities for establishing the genetic counselor profession in Vietnam. This approach allowed participants to share in‐depth and nuanced perspectives informed by their unique contexts (Peters and Halcomb 2015; Kallio et al. 2016). Collecting rich qualitative data was essential to understand participants' complex perspectives on genetic counseling services given the limited published evidence on genetic counselor implementation in Vietnam.

To strengthen the actionability of this study's findings, we structured this study around the Consolidated Framework for Implementation Research (CFIR) (Damschroder et al. 2009, 2022), which is extensively used in global health pre‐implementation studies. We used CFIR during the development of our interview guide to explore multilevel implementation factors (Outer Setting, Inner Setting, Individuals, Innovation, and Implementation Process), and after inductive theme development to map findings to implementation domains.

This study received ethics approval (#81294) from the Stanford University Institutional Review Board (IRB) under an exempt review category for minimal‐risk research involving adult professionals.

### 2.2 Participants

We sought to interview healthcare professionals and institutional leaders in Vietnam who currently provide, oversee, or influence genetics‐related clinical care, testing, research, education, community support, or policy. Participant inclusion criteria followed criterion‐based sampling (Nyimbili and Nyimbili 2024). Participants were eligible if they (1) were affiliated with a Vietnamese institution engaged in genetics‐related clinical care, research, education, or advocacy; (2) held a current or recent role as a healthcare provider, lab director, geneticist, academic faculty member, department head, or leader in a relevant professional or advocacy organization; (3) were ≥ 25 years old; and (4) were willing to participate in a 60‐min interview via Zoom (Zoom Video Communications Inc. 2025) in English or Vietnamese.

We applied structured purposive sampling (Robinson 2023) to recruit participants across Vietnam's healthcare system and used snowball sampling (Parker et al. 2019). To ensure diversity in geography, roles, and institutional types of participants, we initially selected 20 public and private genetics‐related institutions (hospitals, testing laboratories, advocacy organizations, and public health agencies) across Northern, Central, and Southern Vietnam upon which to target study recruitment. Using institutional websites, professional networks, and personal referrals, we identified potential participants and their contact information for outreach. To ensure consistency in outreach, a single researcher (MTB) sent recruitment messages directly to potential participants via email, WhatsApp, Zalo, Facebook Messenger, or LinkedIn (WhatsApp LLC 2025; VNG Corporation 2025; Meta Platforms Inc. 2025; LinkedIn Corporation 2025). To extend the reach of our sample, we invited recipients to forward the invitation to eligible colleagues and requested snowball referrals following interviews.

We screened potential participants using a brief demographic survey via Qualtrics (2023) (Table S1) to verify participant eligibility and monitor sample composition during recruitment. We used survey responses to guide outreach to individuals from institutions and institutional types that were not yet represented, aiming to include 1–2 respondents per institution and achieve informational representativeness (Sandelowski 1995) across institutional types, regions, and healthcare roles. No eligible respondents who completed the demographic survey were excluded. We intentionally sought to balance perspectives from public and private healthcare, genetics, and academic institutions to reflect differences in resource availability, training and familiarity with genetics and genetic counseling, and socioeconomic status of patients whom participants support.

The sample size of 25 was guided by principles of information power (Malterud et al. 2016), which recognizes that studies with focused aims, a sample that is specific, and rich interview data may require fewer participants for meaningful insights. Recruitment concluded once the data were judged to be sufficiently information‐rich to support interpretive depth across target participant groups.

### 2.3 Data Collection

Between July and December 2025, interviews were conducted remotely via Zoom in English or Vietnamese, based on participant preference, with a single interviewer (MTB) who utilized Stanford‐affiliated Vietnamese interpreters as requested.

Four members of the research team (MTB; KEO; PJA; BM) collaboratively developed the semi‐structured interview guide (Table S2). We acknowledge that each of us brings distinct positionalities shaped by Western‐based training and varied international and local experience that informed this process and subsequent analytic interpretation. MTB (she/her), a Master's student in Genetic Counseling at Stanford University and a native of Ho Chi Minh City, Vietnam (dual U.S./Vietnamese citizen; fluent in English, proficient in Vietnamese), draws on prior experiences in Vietnamese public and private oncology settings to bring cultural sensitivity to data analysis, while acknowledging that this also shaped her expectations about unmet needs in Vietnam. KEO (she/her), a U.S.‐trained genetic counselor and global genetic counseling researcher who currently lives and works in Europe, acknowledges the Western background of her training, yet centers her work on equitable global access to genetic services. PJA (he/him), a Philippine‐trained genetic counselor and nurse from Manila who is completing a PhD in the U.S., draws on regional experience building culturally tailored genetic counseling in the Philippines and across Asia. BM (she/her) is a French‐trained medical oncologist with a Master's in Human Genetics and direct clinical experience in France and Vietnam. Together, our positionalities combine Western models of genetic counseling with regional LMIC implementation insight and Vietnam‐based clinical realities–perspectives we drew on reflexively throughout.

We developed targeted questions informed by a literature review of genetic counseling implementation in Asia and other LMICs, and structured the guide using CFIR. Questions were tailored by participant role and covered five key domains: participant knowledge of genetic counseling, system readiness and infrastructure, opportunities and unmet needs, implementation barriers, and sociocultural considerations. We piloted the interview guide on the first participant, feedback from whom informed small revisions to the wording of questions and probes.

With consent, we securely recorded and transcribed interviews using Zoom's transcription software in both English and Vietnamese. A single researcher (MTB) cleaned and prepared bilingual transcripts to ensure verbatim accuracy and de‐identification. Any portions of interviews that were conducted in Vietnamese were subsequently translated into English using DeepL (2025), which the interviewer (MTB), who is proficient in Vietnamese, carefully reviewed to ensure preservation of meaning and nuance.

Data collection and analysis occurred iteratively. While the interview guide remained mostly stable throughout data collection, early analytic memoing and coding informed minor refinements to follow‐up prompts, acquiring more depth and clarification on developing themes in subsequent interviews.

### 2.4 Data Analysis

To iteratively develop themes while acknowledging the researcher's role in meaning‐making, we used inductive Reflexive Thematic Analysis (RTA; Braun and Clarke 2024). In accordance with RTA, data analysis followed six phases: familiarization with the data, initial coding, theme development, theme review, theme definition, and final interpretation.

We imported transcripts into Dedoose (SocioCultural Research Consultants LLC 2026) for data management and coding. A single researcher (MTB) conducted initial data familiarization by reading cleaned and translated transcripts, while writing analytic memos to document impressions, emerging patterns, and reflexive notes (Braun and Clarke 2024).

A single researcher (MTB) then inductively developed a preliminary set of codes, which was piloted on a subset of five transcripts. The preliminary codes were refined through peer debriefing (Ahmed 2024) and collaborative coding discussions with a co‐coder (AMR) and three members of the research team (KEO, PJA, BM). We used collaborative coding to develop a shared understanding of the codes and enrich interpretation with multiple interpretive perspectives (Coulston et al. 2025). Through this process, we reorganized and consolidated codes, clarified code definitions, and refined inclusion and exclusion criteria to support consistency across the dataset. Three researchers (MTB, AMR, KEO) conducted two rounds of collaborative coding, and the codes continued to evolve during full coding. Thereafter, a single researcher (MTB) iteratively refined code definitions as interpretation deepened during full coding, documenting each revision in the audit trail (Giesen and Roeser 2020).

To enrich interpretation and refine the application of codes, the same subset of 20% of transcripts (five interviews) was coded by both a single researcher (MTB) and a co‐coder (AMR). Coders met weekly to compare code applications and discuss divergences to deepen interpretation. We documented decision rules and reflexivity notes throughout this process to support interpretive depth for the remainder of the transcripts.

Once coding was complete, a single researcher (MTB) reviewed the coded excerpts to construct higher‐order patterns. Dedoose tools, such as co‐occurrence patterns and descriptor‐by‐code matrices, supported the iterative development and organization of themes. After we inductively developed themes, a single researcher (MTB) mapped final themes to CFIR domains to ground findings in an implementation‐focused framework. The entire research team discussed the themes for collaborative theme refinement and interpretive depth. Additional strategies to support trustworthiness, informed by Lincoln and Guba (1985), are described in the Supporting Information.

## 3 Results

We interviewed 25 healthcare professionals, institutional leaders, and genetics specialists representing diverse institutional settings, professional roles, and geographic regions across Vietnam (Table 1). This sample reflects perspectives across multiple levels of the healthcare system–from patient‐facing clinicians to community advocates, laboratory, research, and policy professionals–all of whom are essential for the successful integration of a new healthcare profession.

**TABLE 1:** Demographic and professional characteristics of study participants (*N* = 25).

Characteristic	*n* (%)
Self‐described gender	
Woman	12 (48)
Man	13 (52)
Years in current profession	
< 5 years	1 (4)
5–10 years	11 (44)
11–20 years	6 (24)
> 20 years	7 (28)
Country of highest educational training	
Vietnam	20 (80)
Outside Vietnam	5 (20)
Formal genetics training	
No or uncertain formal genetics/genetic counseling (GC) training	12 (48)
Formal training in both genetics and GC	5 (20)
Formal genetics training only	5 (20)
Formal GC training only	3 (12)
Self‐assessed familiarity with the genetic counselor profession^a^	
Aware only	5 (20)
Somewhat familiar	6 (24)
Familiar	9 (36)
Very familiar	5 (20)
Professional role	
Geneticist/Genetic Counselor	4 (16)
Physician—Pediatrics	4 (16)
Physician—Obstetrics/Gynecology (OB/GYN)	3 (12)
Physician—Oncology	3 (12)
Physician—Other specialties^b^	3 (12)
Clinical leadership	3 (12)
Advocacy organization leadership	2 (8)
Testing laboratory leadership	1 (4)
Genetics/genomics researcher	1 (4)
Public health/policy advisor	1 (4)
Clinical population(s) served (select all that apply)^c^	
Pediatrics	11 (19)
Prenatal/reproductive care	8 (14)
Preventive screening	8 (14)
Rare/undiagnosed conditions	7 (12)
Oncology/hematology	7 (12)
Neurogenetic/neurodevelopmental disorders	6 (10)
Genetic testing/referral services	5 (9)
Other clinical populations^d^	3 (5)
Not patient‐facing	3 (5)
Frequency of genetics‐related encounters	
Frequently (weekly or more)	10 (40)
Regularly (monthly or more)	10 (40)
Occasionally (a few times per year)	5 (20)
Socioeconomic background of populations served (select all that apply)^c^	
Primarily private‐pay/higher‐income	21 (46)
Public insurance/charity/lower‐income	13 (28)
Patients from rural areas	12 (26)
Primary organization type	
Public hospital	9 (36)
Private hospital/clinic	8 (32)
Advocacy/NGO	3 (12)
Genetic testing lab	2 (8)
Global healthcare/industry	1 (4)
Government/public health agency	1 (4)
Specialty genetics center/institute	1 (4)
Primary workplace region	
South Vietnam	15 (60)
North Vietnam	6 (24)
Central Vietnam	4 (16)

^a^ Categories correspond to response options for the question “How familiar are you with the profession of genetic counselors?” A fifth option, “Never heard of it before,” was offered but selected by no participants and is not shown. “Aware only” reflects having heard of genetic counselors without knowing what they do; “Somewhat familiar,” some familiarity with what they do; “Familiar,” an understanding of their role; and “Very familiar,” an understanding of their role together with prior interaction or collaboration.

^b^ Other specialties include hematology, rare disease, and internal medicine.

^c^ Multiple selections permitted per participant response. For multi‐select categories, n reflects the number of selections, and percentages are calculated from the total number of responses rather than the number of participants.

^d^ Includes internal medicine, disability services, and infectious disease.

Physician participants represented varied degrees of genetic engagement and supported clinical populations commonly seen in genetic counseling. The sample spanned varied socioeconomic patient populations and represented varied genetics and genetic counseling training and exposure, although all participants reported having genetics‐related encounters.

Interviews lasted an average of 72 min (range: 35–130 min) and were conducted in English (*n* = 8, 32%), Vietnamese (*n* = 6, 24%), or a combination of both (*n* = 11, 44%). From these data, we developed three major themes: (1) genetic testing is outpacing genetics specialists, (2) family, spiritual, and hierarchical frameworks shape the patient response to genetic care, and (3) a phased implementation pathway for genetic counselors is needed. These themes are reflected in Figure 1.

**FIGURE 1:**
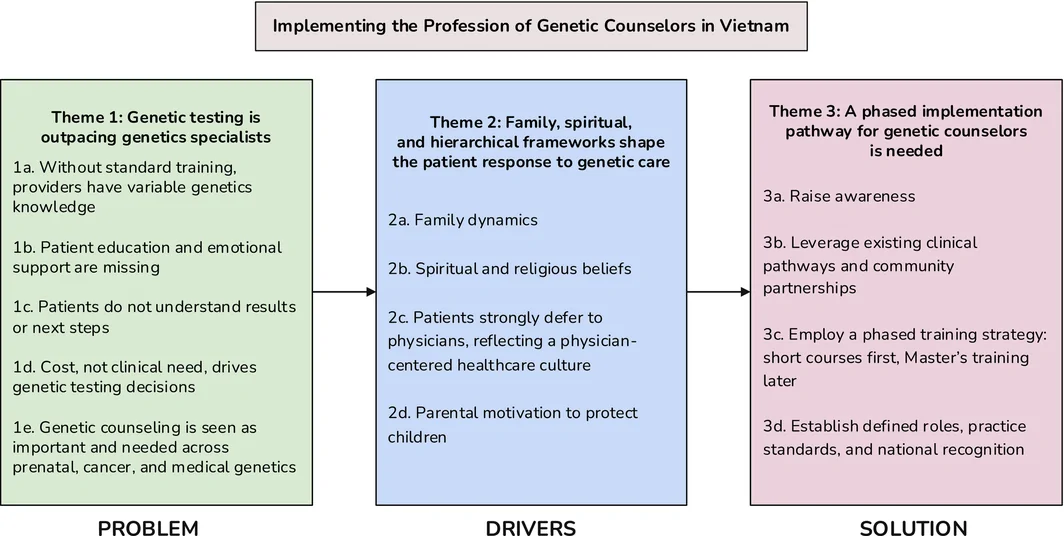
Conceptual model for implementing the profession of genetic counselors in Vietnam. The model synthesizes three themes developed from qualitative interviews with study participants. The problem (Theme 1) and cultural drivers (Theme 2) inform a proposed solution (Theme 3). Participants are identified throughout by numeric code to reduce the risk of reidentification within Vietnam's small genetics and healthcare community.

### 3.1 Theme 1: Genetic Testing Is Outpacing Genetics Specialists

Participants described a widening gap between the rapid growth of genetic testing and the limited availability of trained genetics specialists. This gap undermined clinician confidence and counseling quality, limited translation of test results into action and follow‐up, and made testing decisions more cost‐driven than clinically guided–underscoring the need for genetic counselors across specialties.

Without a standard training pathway, participants who are providing genetic counseling reported learning genetic content, such as genetic testing and result interpretation, through a variety of modalities, including self‐study, short courses, company‐run workshops, and overseas‐trained mentors. Many clinicians felt they lacked confidence in ordering or explaining results, even when they saw the benefits of providing genetic counseling services.

[Learning to provide] genetic counseling was very difficult for me, because I mainly had to self‐study… And the more I did it, the more I felt that genetic counseling is very meaningful for patients. P7 (public medical geneticist)

The challenge of result interpretation was most pronounced with uncertain results. Some participants admitted previously misinterpreting variants of uncertain significance (VUS) results as pathogenic, and they stressed the difficulty in explaining VUS to patients. The lack of a national variant database compounds this challenge, leaving physicians to rely on international databases that do not reflect Vietnam‐specific allele frequencies, which amplifies the difficulties.

I used to… be very confused about the VUS, especially in the patient with already cancer… I will [treat] the patient like they have the [pathogenic] mutation… most of them [do] not clearly understand [VUS results], because it's hard… to explain what it means to the patient. P1 (private oncologist)

These clinician knowledge gaps, combined with limited time and few trained genetics specialists, mean that key counseling components like patient education and emotional support are limited. This need for genetic counselors is most acute in public hospitals, where physicians describe seeing 50–100 patients daily; with a shortage of genetics specialists, they often provide unpaid genetic counseling outside clinical hours.

Doctors are typically focused on treatment, so the time they can devote to counseling‐related issues is quite limited… One or two appointments are often not enough… it requires a longer process, with ongoing conversations and sustained communication… Therefore, I think that having dedicated specialists, [genetic] counselors who can spend more time with patients, is extremely necessary… P24 (private oncologist and clinical leader)

Participants consistently described that while patients currently undergo genetic testing in Vietnam, their test results rarely lead to informed actions. Many participants expressed that patients leave posttest counseling without understanding their results or next steps for treatment, reproductive options, risk reduction, or family communication. Unclear accountability among physicians regarding next steps means that follow‐up and cascade testing are frequently missed.

After that explanation, the patient is not clearly understand what it means, and also how they will follow up with their mutation… how to inform the family members… We usually face the patient with breast cancer… they found they have BRCA mutation… [The] primary doctor there will focus on follow‐up on the patient. We will miss the family. No one will do the cascade [testing]… P1 (private oncologist)

Compounding all of these gaps is cost, often creating barriers before counseling can even take place. Genetic testing in Vietnam is paid entirely out of pocket, typically costing between 7.5 and 15 million Vietnamese dong.^1^ Participants described that cost is often the rate‐limiting step for families; this expense and its feasibility determine the breadth and type of testing. When testing is unaffordable, families may never receive a diagnosis or recurrence risk counseling; as such, some suffer repeated adverse pregnancy outcomes and multiple children born with the same potentially preventable condition.

Everything in this context is out‐of‐pocket… There are families who simply cannot afford it… They end up more or less giving up… There are some patients who have experienced four or five miscarriages. It is really heartbreaking… P19 (public OB/GYN)

After reflecting on these gaps in follow‐up, provider knowledge, emotional support, and time, participants expressed a need for genetic counselors across all core genetic counseling specialties.

I think [genetic counselors are] actually very necessary… When it comes to having in‐depth conversations with the family about the meaning of that mutation, and providing counseling for future generations, and most importantly, addressing the psychological and emotional aspects for the family, clinical physicians are not able to do that. P17 (public pediatrician)

Each specialty is ordering genetic tests, and each carries distinct unmet needs. Prenatal care was highlighted as an area with high demand to prevent genetic conditions in future children and reduce family burden. Needs in oncology and medical genetics were highlighted for targeted cancer treatment, cascade testing, and emotional and clinical complexity for diagnosis, management, and prevention.

### 3.2 Theme 2: Family, Spiritual, and Hierarchical Frameworks Shape the Patient Response to Genetic Care

Participants described a variety of sociocultural factors that add to the complexity of providing genetic counseling services in Vietnam. Genetic information is received not by individual patients, but rather within families, spiritual explanatory frameworks, and hierarchical clinical relationships that shape how patients understand and act on it.

Firstly, family dynamics, including blame, shame, and gendered expectations, shape how patients receive and respond to genetic information. Participants described families and physicians alike blaming mothers for genetic conditions in children, while patriarchal pressure to bear sons sometimes overrode medical recommendations surrounding genetic risk, especially in lower‐income families.

She very much wants to have a son, because she needs to maintain her husband's family line… she is not able to pursue options such as embryo screening or IVF, because she does not have the financial means… she went on to have four children… all four children had hyper‐IgM syndrome, and all of them died very early. P22 (public pediatrician)

Shame and concerns for discrimination also discouraged patients from sharing results and pursuing cascade testing within their families.

They want to hide it from the family, or they want to hide from… the partner… if they have any risk of genetic mutations… They feel shame, they feel that will not be good for them, and that will be not good for the family or their partner…. That also comes from… the Vietnamese culture. P25 (private OB/GYN and clinical leader)

Resistance to cascade testing also came from a cultural tendency to prioritize immediate health concerns over future risk, especially among asymptomatic relatives.

They look today, and they don't think far [ahead]. They are… much more [focused] on taking care… of the [current] problem, [rather] than preventing problems… They believe a lot in luck… if they have good luck, why [do] I need to spend money? And if I have bad luck, well, we'll see when it happens. P4 (private internal medicine physician)

Most [Vietnamese people] will hesitate to do the genetic testing… they want to live happily without knowing the future… When you tell someone… to go to the hospital to do [a] checkup, most of the time they will refuse because they are asymptomatic… It's more acting [reactive] than prevent[ative]. P1 (private oncologist)

Secondly, spiritual and religious beliefs add another layer of complexity that shapes patient responses to genetic care. Many participants felt spiritual beliefs, including fate, luck, or astrology, sometimes override genetic explanations. Religious beliefs often create additional challenges in cases involving termination and predictive testing.

[If] their ages or astrological signs are considered incompatible, families may attribute the child's condition to those factors rather than to scientific causes… they may not accept pregnancy termination… it becomes especially necessary to have someone who can counsel the family… P17 (public pediatrician)

Thirdly, Vietnam's physician‐centered healthcare culture creates complexity that shapes who patients trust and what they expect from genetic care. Participants described that patients often unconditionally trust their physicians, with most expecting one provider to manage all aspects of their care and not distinguishing between clinical specialties.

In Vietnam… the one that patient trusts the most is doctor… they still think any doctor [of any specialization] is the same… they [do] not really know that [a] genetics doctor is really different [from a] pediatric doctor. P10 (public health official/policy advisor)

Finally, not all sociocultural factors that participants identified were barriers–parental motivation was highlighted as a potential facilitator of genetic counseling uptake. Participants agreed that Vietnamese parents, especially mothers, are profoundly motivated to protect their children's health and are receptive to preventative genetic guidance. Several proposed prenatal genetics as a strategic entry point for genetic counselor implementation.

In Vietnam, the anxiety of the mothers to try to do everything… to make sure that the pregnancy and the baby will be okay. So if somebody is going to listen to an advice to prevent a condition, it's going to be the mothers.

P4 (private internal medicine physician)

### 3.3 Theme 3: A Phased Implementation Pathway for Genetic Counselors Is Needed

Participant responses converged on a roadmap for how best to establish the profession of genetic counselors in Vietnam: raise awareness, leverage existing infrastructure, develop training pathways, and establish regulatory frameworks.

Participants identified raising awareness of genetic counseling, both as an available service and for its role and value in care, as the essential first step. Greater clarity is needed among clinicians and the broader public about what genetic counselors do, what the process of genetic counseling entails, and when and how it can help.

There is a real need to apply genetic counseling in Vietnam, and also a real need to educate doctors and the community. P16 (genetics researcher and laboratory leadership)

Many participants independently cited the once‐niche fields of nutrition and psychology in Vietnam, expressing that once the clinical value of genetic counseling is demonstrated, the field could follow the successful trajectory of these professions, which are now nationally recognized and reimbursed.

The national insurance covers for the nutrition[al] counseling, for the psychological counseling… I think the same situation will apply for the medical genetic counseling. P13 (public pediatrician)

Extending the analogy, some participants anticipated that with national recognition and formal training in place, students from related backgrounds would enroll, eventually building a workforce distinct from already overextended physicians.

The second implementation step participants recommended was embedding genetic counselors within established clinical and patient‐support pathways. They agreed that successful implementation will rely on multidisciplinary collaboration and referrals from physicians in obstetrics, pediatrics, oncology, and from genetic testing labs for shared promotion and streamlined logistics.

There needs to be very close collaboration between physicians and genetic counseling specialists. This collaboration helps patients begin to see the necessity and value of genetic testing and genetic counseling. Once trust has been established… genetic counseling could gradually become an independent profession. P24 (private oncologist and clinical leader)

Beyond clinical pathways, participants identified patient support organizations as potential partners for genetic counselors. Given the clinical gaps patients face in emotional and social support, many are turning to these groups for help, often via social media like Facebook. However, participants described these support organizations as lacking the genetics and counseling expertise needed to meet patients' needs, pushing some patient leaders to seek their own genetic counseling training to support other families. Some participants proposed that genetic counselors can bridge this gap and are positioned to bring genetics and counseling expertise directly into the communities where patients are already seeking support, extending reach, trust, and referrals for genetic counseling services.

We know that they [patient support groups] exist, and we know they work for other families… [but] we [clinicians] don't have a good connection with them. P13 (public pediatrician)

I trained [a mother of a child with Duchenne muscular dystrophy] to help me to explain about the genetics, genetic testing, and genetic disorder, and help the other parents to understanding about the emotional burden when they receive the genetic testing report and receive a diagnosis… Because we don't have enough resources to help the patients. P9 (medical geneticist and patient advocate)

Third along the implementation roadmap, participants suggested a phased training strategy to build Vietnam's genetic counselor workforce. In the near term, short courses can upskill physicians to provide basic genetic counseling services, meeting immediate needs while building appreciation for the specialty's value and complexity. In the long term, participants stressed the need for a formal Master's program to build a competent workforce. Genetic counseling could start with physician‐led efforts and build towards a stepped‐care model, whereby physicians provide first‐line genetics care and refer more complex cases–such as those with psychosocial distress, multisession family counseling, or high‐stakes reproductive decisions–to genetic counselors as capacity grows.

I think we need both, because Vietnamese doctors don't have enough time to study for two years, but they still have to do this work, so we can only offer short courses to help them fill the gap… We still need training programs that provide systematic, foundational education… we need neutral genetic consulting courses, not affected by the genetic testing companies… P9 (medical geneticist and patient advocate)

Finally, the successful implementation of genetic counselors requires regulatory safeguards and practice standards. Participants highlighted the lack of recognition from the Ministry of Health as a foundational barrier to the development of both the genetic counselor and medical geneticist professions. Without an official title, defined scope, or licensure pathway, clinicians lack the institutional and legal protection to confidently provide and promote genetic counseling services. This, combined with a lack of standardized referrals and follow‐up processes, creates systemic inefficiencies due to blurred roles of geneticists, genetic counselors, and ordering clinicians.

In Vietnam, we are… not have an official definition for genetic counselor and medical geneticist… there's no mention about genetic counselors… When the new regulatory frameworks appear… they can have more chance to work. P9 (medical geneticist and patient advocate)

Participants also stressed a need to develop Vietnam‐specific genetic counseling practice standards, noting that reliance on lengthy foreign guidelines creates barriers in language, time, and local relevance. This contributes to inconsistent practices in genetic care–some clinicians order predictive genetic testing for minors, and patients have limited legally protected preventative options to address genetic risk, such as prophylactic surgeries for hereditary cancer genes.

In Vietnam, we have no guideline… we mainly use the latest guideline of the NCCN. But how I can read it?… It's written by English… [It takes] a lot of time to work, in a busy day. P11 (private medical geneticist)

### 3.4 Implications for the Profession of Genetic Counselors: Mapping to CFIR

The described roadmap proposal for implementing the profession of genetic counselors in Vietnam maps onto all five CFIR domains (Figure 2), suggesting actionable next steps across multiple levels of the healthcare system. Theme 1–genetic testing is outpacing genetics specialists–maps to Inner Setting constraints (limited time, unclear accountability, and weak follow‐up infrastructure) and Individuals (variable genetics knowledge and confidence). Participants also described strong Innovation, emphasizing a clear perceived fit and specialty‐specific needs for genetic counselors across cancer, prenatal, and medical genetics settings. Theme 2–family, spiritual, and hierarchical frameworks shape the patient response to genetic care–maps primarily to the Outer Setting, highlighting how familial, religious, and healthcare norms should shape service and training design. Theme 3–a phased implementation pathway is needed–maps to Implementation Process, outlining practical steps spanning both Inner Setting (training and workflow integration) and Outer Setting changes (professional recognition and national guidelines). Together, this CFIR‐mapped guide provides participant‐identified needs and recommendations at every ecological level for improved genetic counseling practice, education, and policy in Vietnam.

**FIGURE 2:**
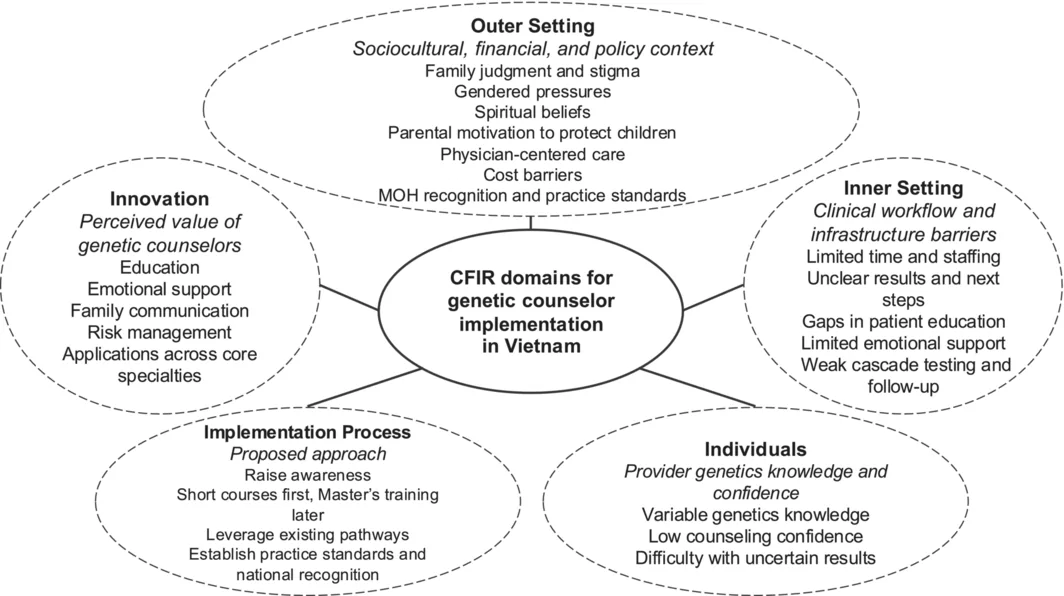
CFIR Mapping of genetic counselor implementation in Vietnam. Study findings from qualitative interviews mapped onto the five domains from the Consolidated Framework for Implementation Research (CFIR) (Damschroder et al. 2022). MOH, Ministry of Health.

## 4 Discussion

This study provides the first needs assessment for the profession of genetic counselors in Vietnam. Through 25 semi‐structured interviews with healthcare leaders and genetics professionals across Northern, Central, and Southern Vietnam, we developed three major themes: genetic testing is outpacing genetics specialists; family, spiritual, and hierarchical frameworks shape the patient response to genetic care; and a phased implementation pathway for genetic counselors is needed. These findings highlight both the urgent need and a practical pathway for establishing the profession of genetic counselors in Vietnam, and offer insights to support the development of the profession of genetic counselors in other LMICs around the world.

Our study findings reflect the broader global pattern of a widening “genomics divide” (Pang and Weatherall 2002, 1051), where genetic sequencing technology is outpacing the specialists and the systems needed to apply it (Jenkins et al. 2021). In Vietnam, participants similarly described patients leaving testing without a clear understanding, limited emotional support, and missed cascade testing for at risk relatives. Participants attributed these gaps to a lack of time and standardized genetics training for clinicians and difficulty accessing and translating non‐Vietnamese professional guidelines, leading to variable genetics knowledge, low counseling confidence, and particular difficulty navigating uncertain results–patterns also reported in other settings with limited genetic counseling (Chou et al. 2021; Osler et al. 2025; Menke et al. 2021). The lack of Vietnam‐specific variant databases amplifies this challenge; Asian patients have been reported to be over twice as likely to get a VUS result in hereditary cancer genes compared to European patients (Ndugga‐Kabuye and Issaka 2019). This underscores the need for Vietnam‐specific variant resources and professional guidelines to minimize potential patient harm from overtreatment and undue anxiety. Finally, cost, rather than clinical need, appears to be driving testing decisions and access–a challenge documented across the Global South (Kwong et al. 2025; Chávarri‐Guerra et al. 2024; Pederson and Narod 2024). Genetic testing in Vietnam is paid entirely out of pocket and can be prohibitively expensive (Toan et al. 2026), often costing an entire 1–2 months of average wages (Viet Nam Social Security 2024), and considerably more for households in rural areas, where average incomes are lower (National Statistics Office of Viet Nam 2026). With testing and genetics expertise concentrated in Hanoi and Ho Chi Minh City, rural and ethnic minority patients additionally face travel, lost wages, and language barriers (McKinn et al. 2017), contributing to worse health outcomes for these already‐disadvantaged populations (Ha et al. 2024). Interpreter services and telehealth delivery models could support equitable access, as they have for remote populations in the Philippines (Padilla et al. 2024).

Our findings also highlight sociocultural factors shaping both the need for and delivery of genetic counseling in Vietnam; these are consistent with dynamics documented across Asian countries with emerging genetic counseling services. For example, fear of discrimination and stigma similarly limit result disclosure in Korea (Jin et al. 2026); cultural son preference and blame towards mothers influence reproductive decision‐making in India and Asian‐American communities (Bowman‐Smart et al. 2020; Gupta 2007; Zhong et al. 2021; Mittman et al. 1998); parents are highly motivated to have healthy children and receptive to prenatal genetic testing in Hong Kong (Ngan et al. 2020); and deference to physician authority may facilitate greater genetic counseling uptake when recommended by a doctor in Indonesia, Singapore, and Vietnamese‐American communities (Claramita et al. 2011; Chieng et al. 2011; Lemke et al. 2022). Participants' references to fate, luck, and astrology reflect Buddhist and folk religious frameworks widely drawn on in Vietnam—ethnographic studies find Vietnamese patients often understand congenital conditions as karmic consequences, implicating the parents' or ancestors' moral narratives rather than the child's biology alone (Gammeltoft 2008; Ngo et al. 2012; Ha et al. 2014). Beliefs about karma, fate, and spiritual punishment similarly shape how families understand recurrence risks and explain genetic conditions in Thailand and the Philippines (Zayts et al. 2013; Abad et al. 2014), highlighting the need for genetic counselors to elicit and work within each family's explanatory framework. Taken together, these factors increase the complexity of genetic counseling and underscore the need to use family‐centered approaches that engage with these sociocultural frameworks without dismissing them. Strategically, such findings also support positioning prenatal genetic counseling as an implementation entry point and leveraging physician collaboration.

These findings also underscore possible limitations of applying Western‐based genetic counseling models into the Vietnamese context, as these models assume counseling tenets incongruent with the Vietnamese lived realities. For example, the Reciprocal‐Engagement Model, which guides genetic counseling practice in North America, centers individual autonomy and nondirectiveness (Veach et al. 2007), clashing with what our participants described, where families decide collectively, and patients often desire directive healthcare (Chieng et al. 2011; Zayts et al. 2013). It is in this context that the World Health Organization (2022), and more recently Weil et al. (2024), proposed that effective global genetic counseling must be co‐designed with local communities, and tailored to local sociocultural norms and values. For Vietnam, this means co‐developing the profession's implementation and training with Vietnamese clinicians, patients and families, patient support organizations, and health system leaders, grounded in the sociocultural frameworks documented here and elsewhere, rather than simply replicating Western norms of genetic counseling. In the Philippines, Abad et al. (2026) demonstrate an alternative that builds outward from indigenous relational concepts to reframe the counselor‐client relationship rooted in Filipino lived realities. Similar efforts in Vietnam will contribute to the growing movement to decolonize genetic counseling by developing frameworks that recenter the lived realities of the communities genetic counselors serve (Weil et al. 2024; Abad et al. 2026). In doing so, Vietnam will not only better serve its population but also offer a replicable model of culturally grounded genetic counselor implementation for LMICs worldwide.

In our study, participants converged on a roadmap for establishing the field of genetic counselors in Vietnam. By mapping our study results to every CFIR domain, our findings point to staged implementation priorities: build awareness and referral pathways; embed services into existing prenatal, oncology, and pediatric pathways and community networks; develop a phased training strategy beginning with short courses for physicians while developing Master's training for genetic counselors; and in the long term, establish Ministry of Health recognition, scope, and national guidelines—a roadmap which mirrors successful implementation pathways in other Southeast Asian countries (Laurino et al. 2017; Cutiongco‐de la Paz et al. 2019). Southeast Asian models represent different stages of genetic counselor professionalization. Singapore, for instance, has embedded Master's‐trained genetic counselors within clinical care, but has not yet established a local formal training program (Lim et al. 2025). Meanwhile, the Philippines has established a genetic counseling training pathway, but is still striving towards regulatory recognition (Lim et al. 2025). Thus, this roadmap may need to proceed flexibly in Vietnam, adapting to local systems and priorities, rather than in a strict linear sequence.

Participants highlighted building awareness as the first stage of professionalization. As physicians serve as the trusted gatekeepers for referrals, low provider awareness can lead to missed referrals, inappropriate test ordering, and limited patient understanding (Lim et al. 2025). Pakistan and the Philippines offer models for successful genetics education campaigns (Furqan et al. 2024; Laurino and Padilla 2013). Consistent with patterns of successful professionalization across Asia and the Global South, participants also recommended embedding genetic counselors within established clinical pathways, multidisciplinary teams, and community groups to leverage existing referral networks and trust (Nakamura et al. 2016; Pacheco‐Cuellar et al. 2020; Laurino and Padilla 2013). In the Philippines, positive newborn screens trigger referral for genetic counseling (Padilla et al. 2024); Vietnam's expanding newborn screening infrastructure could provide a similar entry point (Therrell et al. 2025; Ministry of Health of Vietnam 2026).

For workforce development, participants proposed a phased training approach recommended in prior work across Asia and other LMICs, with short courses for non‐genetics professionals as an interim solution, equipping them with basic genetic counseling scripts, checklists, and referral rules (Ashfaq et al. 2013; Lim et al. 2025; Bucio et al. 2019). However, participants emphasized that short courses alone are insufficient as a long‐term substitute for Master's training, given the advanced genetics and psychosocial competencies required, echoing experiences in other LMICs where short courses led to calls for 2‐year programs (Salehi and Haghani 2014; Ormond et al. 2024). Master's‐level genetic counselor programs already exist in the Philippines, Malaysia, and Indonesia (Cutiongco‐de la Paz et al. 2019), and the global expansion to over 130 training programs reflects a consensus that Master's‐level training is the professional standard for genetic counseling (Ormond et al. 2024), although there are many different paths to arrive at this standard.

Participants repeatedly cited nutrition and psychological counseling as models of professionalization in Vietnam, expecting the field of genetic counselors to follow suit. Over two decades, both fields advanced from international partnerships that helped establish formal training–Vietnam National University‐Vanderbilt collaboration launched the Master's program in clinical psychology (Weiss et al. 2011, 2012), and a Vietnam‐Japan partnership established the first formal dietetics program and a dietitian job code (Japan International Cooperation Agency [JICA] and Ajinomoto Co. Inc. 2016)–to national recognition, with “psychologist” becoming a recognized occupation in 2020 (Prime Minister of Vietnam 2020). Similarly, some participants anticipated that Master's‐level training would attract students from related fields, eventually creating a workforce distinct from already overextended physicians. Likewise in the Philippines, Malaysia, and Indonesia, the genetic counseling workforce consists largely of nonphysician health professionals including nurses, laboratory scientists, and graduates of biology or public health programs (Cutiongco‐de la Paz et al. 2019; Abad, Tumulak, Guerbo, et al. 2024).

Finally, participants emphasized the need for Ministry of Health recognition, role definition, and national practice guidelines. This absence of regulatory infrastructure has produced barriers well documented across the Asia Pacific, including role ambiguity and inconsistent practices among genetic counseling providers (Laurino et al. 2017, 2018) and a reliance on lengthy foreign guidelines that create accessibility challenges in language, time, and clinical relevance (Kwong et al. 2025)–each introducing patient safety risks (National Society of Genetic Counselors 2025; Gradishar and Salerno 2016). Participants' recommendations align with models of successful genetic counselor implementation across Asia, like Taiwan, where national legislation and coverage policies have helped transform genetic services from niche to routine care (Chien et al. 2013).

## 5 Limitations

Although we used structured purposive sampling to recruit participants across a range of roles and institutions, we received single responses from public health and genetics research institutes. The sample was also regionally imbalanced, with more participants from Southern Vietnam (*n* = 15, 60%) than Northern (*n* = 6, 24%) or Central (*n* = 4, 16%), potentially limiting the transferability to these settings. For example, Southern participants may work in contexts with established private genomics infrastructure (Ormiston and Hanson 2023; Gene Solutions 2025), while participants in Northern and Central settings face documented disparities in healthcare staffing, infrastructure, and specialty services (Feng et al. 2024). Therefore, our findings may underrepresent barriers like specialist scarcity and distance to tertiary care. As a qualitative needs assessment, this study was designed to support transferability rather than statistical generalizability. We also note that participants who opted in may have had a greater interest in genetics and workplace support to participate in research, potentially skewing perspectives to be more positive towards genetic counseling.

Our sample also consisted entirely of clinicians, advocacy professionals, and organizational leaders, but did not directly capture patient and family perspectives. As data were collected in 2025, findings reflect a particular moment in Vietnam's rapidly evolving genomic context; implementation conditions may change as genetic technology, regulation, and workforce develop over time. Many participants chose to interview in English despite not being native speakers, which may have limited nuance for those less fluent. Additionally, participants may have offered socially desirable responses aligned with institutional reputations or commercial interests, especially when discussing system gaps. Finally, while we acknowledge and engage with our own positionalities, the first author's positionality as a Vietnamese‐American genetic counseling student may have shaped participant access, the process of interviews, and the interpretive lens.

## 6 Future Directions

This study maps a starting point rather than a final destination. Further research is needed on patient and family perspectives to inform how the future of genetic counseling services is designed. As training is developed, we encourage characterization of the potential pool from which a genetic counselor workforce could be drawn–upskilled physicians, nonphysician healthcare professionals, patient advocates, and students in related fields–to develop effective and sustainable training. Vietnamese providers' views on how international partnerships should contribute to the development of the field of genetic counselors could also help shape the training approaches. Future research should examine how Western genetic counseling models may need to be adapted to Vietnamese family, clinical, and cultural contexts. As practice expands, it will be useful, for example, to describe how families in Vietnam understand and seek information about environmental contributions to congenital anomalies and other clinically relevant topics. Finally, measuring key performance indicators of the outcomes of genetic counseling–cascade testing uptake, time to diagnosis, and cost‐effectiveness–is encouraged to evaluate and guide the field's development.

## 7 Conclusion

Vietnam is in a pivotal period in genomic healthcare, where genetic testing is increasingly used, but genetic results are not reliably translating into informed action or cascade prevention. Patients and families often leave testing with limited understanding, emotional support, and coordinated follow‐up, and clinicians face time constraints, variable genetics training, and limited Vietnam‐specific resources for variant interpretation. These challenges are shaped by Vietnam's sociocultural context, including family pressure, social judgment, spiritual beliefs, and strong deference to physicians, making genetic counseling both more complex and more essential. Tailoring genetic counselor implementation to Vietnam's sociocultural realities, as documented here, is critical to best serve its population. CFIR mapping translates participant input into a staged implementation plan, spanning from Inner Setting clinical workflows and workforce training to Outer Setting policy and financing, informing practical next steps.

The regional momentum in establishing the profession of genetic counselors in neighboring countries offers Vietnam a wealth of wisdom, expertise, and collaborations to leverage in its own development of the field. The growing number of regional genetic counseling societies, such as the Professional Society of Genetic Counselors in Asia (PSGCA), has strengthened academic exchange and knowledge‐sharing (Abad, Tumulak, Yoon, et al. 2024; Ormond et al. 2024), while national societies have advocated for professional recognition and regulation in Indonesia and Malaysia (Swandayani et al. 2021; Allied Health Sciences Division, Ministry of Health Malaysia 2025; Ormond et al. 2024) and laid the foundation for genetic counseling infrastructure in countries such as the Philippines (Abad, Tumulak, Guerbo, et al. 2024). Partnerships beyond the region have already helped develop genetics expertise in Vietnam: a decade‐long Vietnamese‐North American collaboration supported medical genetics education at Hue University of Medicine and Pharmacy (Leppig 2018), consistent with participants' descriptions of learning genetics through overseas‐trained mentors and short courses. These professional genetic counselor networks support training, case‐conferencing, clinical placements, resource development, and policy advocacy across regions, which Vietnam can similarly leverage as it develops its genetic counselor infrastructure. Vietnam is already primed to capitalize on such networks, given its active involvement in regional collaborations such as the 2015 Genetic Counseling Pre‐Conference Workshop (Laurino et al. 2017, 2018).

In sum, grounded in an implementation framework, this study offers the first locally informed steps to guide the effective establishment of genetic counselors in Vietnam and better support patients and families affected by genetic conditions nationwide. Looking ahead, these findings mark a starting point and identify the research priorities that should shape what comes next.

## Author Contributions

**Mai Ly Tran Burke:** conceptualization, methodology, investigation, data curation, formal analysis, project administration, funding acquisition, writing – original draft, writing – review and editing, visualization. **Peter James B. Abad:** methodology, formal analysis, writing – review and editing. **Basma M'Barek:** investigation, resources, writing – review and editing. **Angelica Michelle Raney:** formal analysis, validation, writing – review and editing. **Janine S. Bruce:** methodology, formal analysis, validation, writing – review and editing. **Sylvia Bereknyei Merrell:** methodology, formal analysis, validation, writing – review and editing. **Hetanshi Naik:** supervision, resources, writing – review and editing. **Kelly E. Ormond:** conceptualization, methodology, supervision, formal analysis, project administration, validation, writing – review and editing.

## Ethics Statement

This study received ethics approval (#81294) from the Stanford University Institutional Review Board (IRB) under an exempt review category for minimal‐risk research involving adult professionals. All procedures were conducted in accordance with recognized ethical standards for human subjects research, including the U.S. Federal Policy for the Protection of Human Subjects and the Declaration of Helsinki. Prior to participation in our study, we provided participants with an English or Vietnamese information sheet; participants provided verbal informed consent at the start of each interview. All data were de‐identified and stored securely, and we anonymized any quoted material. No incentives or compensation were offered to participants for being involved in this study. No nonhuman animal studies were performed by the authors for this paper.

## Conflicts of Interest

Kelly E. Ormond declares no financial conflicts of interest and is the current president of the Transnational Alliance of Genetic Counselors. Peter James B. Abad is a Section Editor for the Journal of Genetic Counseling and co‐author of this study. He is excluded from the editorial decision‐making related to the review of this article for publication in the Journal. The remaining authors, Mai Ly Tran Burke, Angelica Michelle Raney, Basma M'Barek, Hetanshi Naik, Sylvia Bereknyei Merrell, and Janine S. Bruce, declare no conflicts of interest.

## Supporting information


**Table S1:** Demographic survey.
**Table S2:** Semi‐structured interview guide.

## Data Availability

This study's data are not publicly available in order to protect the confidentiality of our participants. However, requests for data access, such as for de‐identified interview transcripts, will be considered upon reasonable request to the corresponding author.

## Endnotes

^1^
Approximately US$300–600 (World Bank 2025).
