# Supplementary material for: Needs, Challenges, and Opportunities for Genetic Counselors in Vietnam: A Qualitative Interview Study With Healthcare Leaders and Genetics Professionals
Source: J Genet Couns. 2026 Oct 10;35(5):e70303. doi: 10.1002/jgc4.70303 (PMC13653685; doi:10.1002/jgc4.70303)
Supplement: Supplementary file 1 [file JGC4-35-0-s001.pdf]

Supplemental methods

1. Trustworthiness and Rigor
2. Table S1 - Demographic Survey
3. Table S2 - Semi-structured Interview Guide

1. Trustworthiness and Rigor

We drew on Lincoln and Guba's (1985) framework, where compatible with a constructivist paradigm, to enhance the credibility and transferability of this qualitative study. Throughout data analysis, a single researcher (MTB) maintained a reflexive journal (Lincoln & Guba, 1985); she memoed her assumptions and evolving interpretations, and reflected on how her potential positionality shaped these. She maintained an audit trail (Lincoln & Guba, 1985) that documented research activities, versions of codes, memos, and changes to methodology.

To enrich interpretation through multiple perspectives and support reflexivity, the research committee (MTB; AMR; KEO; PJA) engaged in two rounds of collaborative coding and peer debriefing (Ahmed, 2024) during code development. To deepen interpretive understanding and refine the application of codes, MTB and a co-coder (AMR) coded the same subset of 20% of transcripts (5 interviews) as a basis for collaborative discussion.

We discussed preliminary themes with the entire research committee. We employed negative case analysis (Lincoln & Guba, 1985) to interrogate interpretations, and collaborative theme refinement to contextualize developing themes with demographic variables and team members' positionality.

During data presentation, we applied thick descriptions with rich, contextualized participant quotes to allow readers to consider the transferability of participant perspectives (Lincoln & Guba, 1985).

2. **Table S1. Demographic Screening Survey**

Bilingual (English and Vietnamese) screening survey used to assess eligibility and collect demographic information from potential participants prior to the interview. Eligible respondents must meet the following inclusion criteria: (1) were affiliated with a Vietnamese institution engaged in genetics-related clinical care, research, education, or advocacy; (2) held a current or recent role as a healthcare provider, lab director, geneticist, academic faculty member, department head, or leader in a relevant professional or advocacy organization; (3) were ≥25 years old; and (4) were willing to participate in a a 60-minute interview via Zoom in English or Vietnamese.

Q#	Survey Questions	Vietnamese Translation
Introduction Message	Welcome, and thank you for your interest in our research!<br><br>Please review the Information Sheet (in English or Vietnamese), which explains the study's purpose, what participation involves, and how your data will be handled.<br>If selected to interview, you'll be asked to provide verbal consent at the start of the interview.<br>Please do not include any personal or patient-identifying information (such as full names, birth dates, or medical record numbers) in your responses.	Chào mừng và cảm ơn bạn đã quan tâm đến nghiên cứu của chúng tôi!<br><br>Vui lòng xem qua Phiếu thông tin (bằng tiếng Anh hoặc tiếng Việt), trong đó giải thích mục đích của nghiên cứu, những nội dung liên quan đến việc tham gia và cách dữ liệu của bạn sẽ được xử lý.<br>Nếu được chọn phỏng vấn, bạn sẽ được yêu cầu cung cấp sự đồng ý bằng lời nói khi bắt đầu cuộc phỏng vấn.<br>Vui lòng không đưa bất kỳ thông tin cá nhân hoặc thông tin nhận dạng bệnh nhân nào (như họ tên đầy đủ, ngày sinh hoặc số hồ sơ y tế) vào câu trả lời của bạn.
1	Do you work in Vietnam? (yes/no)	Bạn có làm việc ở Việt Nam không? (có/không)
2	Job title/role<br>☐ Physician – Oncologist<br>☐ Physician – OB/GYN<br>☐ Physician – Pediatrician<br>☐ Physician – Other<br>☐ Department Head / Clinical Unit Leader<br>☐ Geneticist or Genetic Counselor<br>☐ Laboratory Director / Lab Manager<br>☐ Genetic Testing Specialist / Scientist<br>☐ University Faculty<br>☐ Educational Program Director or Coordinator	Chức danh/vai trò công việc<br>☐ Bác sĩ – Bác sĩ ung thư<br>☐ Bác sĩ – Sản phụ khoa<br>☐ Bác sĩ – Bác sĩ nhi khoa<br>☐ Bác sĩ – Khác<br>☐ Trưởng khoa/Trưởng đơn vị lâm sàng<br>☐ Nhà di truyền học hoặc Cố vấn di truyền<br>☐ Giám đốc phòng xét nghiệm/Quản lý phòng xét nghiệm<br>☐ Chuyên gia/Nhà khoa học xét nghiệm di truyền

	☐ Public Health Official / Policy Advisor<br>☐ Leader / Staff Member – Professional Association<br>☐ Leader / Staff Member – Advocacy or Community-Based Organization<br>☐ Researcher – Genetics or Genomics<br>☐ I am none of these	☐ Khoa đại học<br>☐ Giám đốc hoặc Điều phối viên chương trình giáo dục<br>☐ Cán bộ y tế công cộng/Cố vấn chính sách<br>☐ Lãnh đạo/Nhân viên – Hiệp hội chuyên nghiệp<br>☐ Lãnh đạo/Nhân viên – Tổ chức vận động hoặc cộng đồng<br>☐ Nhà nghiên cứu – Di truyền học hoặc Di truyền học<br>☐ Tôi không phải là bất kỳ ai trong số những người này
3	Are you currently at least 25 years old?<br>☐ Yes<br>☐ No	Hiện tại bạn có ít nhất 25 tuổi không?<br>☐ Có<br>☐ Không
4	Do you agree to participate in a 60-minute research interview about genetic counseling services in Vietnam?<br>☐ Yes<br>☐ No	Bạn có đồng ý tham gia phỏng vấn nghiên cứu 60 phút về dịch vụ tư vấn di truyền tại Việt Nam không?<br>☐ Có<br>☐ Không
5	Location of your primary workplace<br><br>☐ Hanoi<br><br>☐ Ho Chi Minh City<br><br>☐ Central region (e.g., Hue, Da Nang)<br><br>☐ Remote (online)<br><br>☐ Other: [Free text]	Vị trí nơi làm việc chính của bạn<br><br>☐ Hà Nội<br><br>☐ Thành phố Hồ Chí Minh<br><br>☐ Khu vực miền Trung (ví dụ: Huế, Đà Nẵng)<br><br>☐ Làm việc từ xa (trực tuyến)<br><br>☐ Khác: [Văn bản tự do]
6	Primary workplace or institution<br><br>☐ Public hospital<br>☐ Private hospital or clinic<br>☐ Genetic testing laboratory	Nơi làm việc hoặc tổ chức chính<br><br>☐ Bệnh viện công<br>☐ Bệnh viện hoặc phòng khám tư<br>☐ Phòng xét nghiệm di truyền

	☐ University or academic institution<br>☐ Research institute or genetics-focused center<br>☐ Government or public health agency<br>☐ Advocacy or community-based organization<br>☐ Professional association<br>☐ Other (please specify): [Free text]	☐ Trường đại học hoặc tổ chức học thuật<br>☐ Viện nghiên cứu hoặc trung tâm tập trung vào di truyền<br>☐ Cơ quan y tế công cộng hoặc chính phủ<br>☐ Tổ chức vận động hoặc cộng đồng<br>☐ Hiệp hội chuyên môn<br>☐ Khác (vui lòng nêu rõ): [Văn bản tự do]
7	Years of experience in your current profession<br><br>☐ Less than 5 years<br><br>☐ 5–10 years<br><br>☐ 11–20 years<br><br>☐ More than 20 years	Số năm kinh nghiệm trong nghề hiện tại của bạn<br><br>☐ Dưới 5 năm<br><br>☐ 5–10 năm<br><br>☐ 11–20 năm<br><br>☐ Trên 20 năm
8	Gender<br>☐ Woman<br><br>☐ Man<br><br>☐ Prefer not to say<br><br>☐ Prefer to self-describe: [Free text]	Giới tính<br>☐ Phụ nữ<br><br>☐ Đàn ông<br><br>☐ Không muốn nói<br><br>☐ Thích tự mô tả: [Văn bản tự do]
9	In what country did you complete your highest degree or level of education?<br><br>☐ Vietnam<br><br>☐ France<br><br>☐ UK<br><br>☐ USA<br><br>☐ Other (please specify): [Free text]<br><br>☐ Prefer not to say	Bạn đã hoàn thành bằng cấp hoặc trình độ học vấn cao nhất của mình ở quốc gia nào?<br><br>☐ Việt Nam<br><br>☐ Pháp<br><br>☐ Anh<br><br>☐ Khác (vui lòng nêu rõ): [Văn bản tự do]<br><br>☐ Không muốn nói

10	Which of the following best describes the primary clinical population you work with? (Select all that apply)<br>☐ Pregnant individuals (Prenatal care)<br>☐ Children (Pediatric)<br>☐ Oncology patients<br>☐ Adult patients with rare or undiagnosed diseases<br>☐ Individuals with neurodevelopmental disorders (e.g., autism, intellectual disability)<br>☐ Healthy individuals seeking preventive screening<br>☐ Patients referred for genetic testing<br>☐ I do not directly serve patients (please briefly describe your role): ____________<br>☐ Other: [Free text]	Câu nào sau đây mô tả tốt nhất về nhóm dân số lâm sàng chính mà bạn làm việc cùng? (Chọn tất cả các câu trả lời phù hợp)<br>☐ Cá nhân mang thai (Chăm sóc trước khi sinh)<br>☐ Trẻ em (Nhi khoa)<br>☐ Bệnh nhân ung thư<br>☐ Bệnh nhân trưởng thành mắc các bệnh hiếm gặp hoặc chưa được chẩn đoán<br>☐ Cá nhân mắc các rối loạn phát triển thần kinh (ví dụ: tự kỷ, khuyết tật trí tuệ)<br>☐ Cá nhân khỏe mạnh tìm kiếm sàng lọc phòng ngừa<br>☐ Bệnh nhân được giới thiệu để xét nghiệm di truyền<br>☐ Tôi không trực tiếp phục vụ bệnh nhân (vui lòng mô tả ngắn gọn vai trò của bạn): ____________<br>☐ Khác: [Văn bản tự do]
11	Which of the following best describes the types of patient populations you commonly serve based on healthcare access or socioeconomic background? (Select all that apply)<br>☐ Patients from rural areas<br>☐ Patients from high-income backgrounds (private pay)<br>☐ Patients from low-income backgrounds (public insurance or charity-based care)<br>☐ Other: [Free text]	Câu nào sau đây mô tả tốt nhất các loại nhóm bệnh nhân mà bạn thường phục vụ dựa trên khả năng tiếp cận dịch vụ chăm sóc sức khỏe hoặc hoàn cảnh kinh tế xã hội? (Chọn tất cả các câu trả lời phù hợp)<br>☐ Bệnh nhân từ vùng nông thôn<br>☐ Bệnh nhân từ hoàn cảnh thu nhập cao (tự trả)<br>☐ Bệnh nhân từ hoàn cảnh thu nhập thấp (bảo hiểm công hoặc chăm sóc từ thiện)<br>☐ Khác: [Văn bản tự do]
12	How frequently do you encounter patients or cases involving genetic conditions or testing in your work?<br><br>☐ Rarely or never<br><br>☐ Occasionally (a few times per year)<br><br>☐ Regularly (monthly or more)	Bạn thường xuyên gặp phải bệnh nhân hoặc trường hợp liên quan đến tình trạng di truyền hoặc xét nghiệm trong công việc của mình như thế nào?<br><br>☐ Hiếm khi hoặc không bao giờ<br><br>☐ Thỉnh thoảng (vài lần mỗi năm)

	☐ Frequently (weekly or more)	☐ Thường xuyên (hàng tháng hoặc nhiều hơn)<br><br>☐ Thường xuyên (hàng tuần hoặc nhiều hơn)
13	Have you had any formal training in genetics or genetic counseling?<br><br>☐ Yes, genetics only<br><br>☐ Yes, genetic counseling only<br><br>☐ Yes, both genetics AND genetic counseling<br><br>☐ No<br><br>☐ Not sure	Bạn đã được đào tạo chính thức về di truyền học hoặc tư vấn di truyền chưa?<br><br>☐ Có, chỉ di truyền học<br><br>☐ Có, chỉ tư vấn di truyền<br><br>☐ Có, cả di truyền học VÀ tư vấn di truyền<br><br>☐ Không<br><br>☐ Không chắc
14	How familiar are you with the profession of genetic counselors?<br><br>☐ Never heard of it before<br><br>☐ Heard of it, but don’t know what they do<br><br>☐ Somewhat familiar with what they do<br><br>☐ Familiar and understand their role<br><br>☐ Very familiar and have interacted with or worked with them	Bạn có quen thuộc với nghề tư vấn di truyền không?<br><br>☐ Chưa từng nghe đến<br><br>☐ Đã nghe đến nhưng không biết họ làm gì<br><br>☐ Có phần quen thuộc với công việc của họ<br><br>☐ Quen thuộc và hiểu vai trò của họ<br><br>☐ Rất quen thuộc và đã từng tương tác hoặc làm việc với họ
15	Preferred language for the interview<br><br>☐ Vietnamese<br><br>☐ English<br><br>☐ No preference<br><br>☐ Other: [Free text]	Ngôn ngữ ưa thích cho cuộc phỏng vấn<br><br>☐ Tiếng Việt<br><br>☐ Tiếng Anh<br><br>☐ Không có lựa chọn nào<br><br>☐ Khác: [Văn bản tự do]

16	Please provide the best email OR WhatsApp/Zalo phone number to reach you for scheduling [Free text]	Vui lòng cung cấp email HOẶC số điện thoại WhatsApp/Zalo tốt nhất để liên lạc với bạn để lên lịch [Tin nhắn miễn phí]
17	Please provide your name [Free text]	Họ và tên đầy đủ [Văn bản tự do]
18	Please open this link to enter your upcoming availability for a 1-hour-long interview via Zoom. // [if unable to access the link:] Please contact the study director directly to schedule your interview:<br>Email: maily@stanford.edu	Vui lòng mở liên kết này để nhập thời gian phỏng vấn sắp tới của bạn kéo dài 1 giờ qua Zoom. // [nếu không thể truy cập liên kết:] Vui lòng liên hệ trực tiếp với giám đốc nghiên cứu để lên lịch phỏng vấn:<br>Email: maily@stanford.edu
Concluding Message	Thank you for taking the time to complete this survey.<br>If selected for an interview, the study director will contact you using the details you provided.<br><br>For any questions, feel free to contact maily@stanford.edu.<br><br>If you'd like to review the study details again, the Information Sheet is available here in English or Vietnamese.	Cảm ơn bạn đã dành thời gian để hoàn thành khảo sát này.<br>Nếu được chọn phỏng vấn, giám đốc nghiên cứu sẽ liên hệ với bạn bằng thông tin bạn cung cấp.<br><br>Nếu có bất kỳ câu hỏi nào, vui lòng liên hệ với maily@stanford.edu.<br><br>Nếu bạn muốn xem lại thông tin chi tiết về nghiên cứu, Phiếu thông tin có sẵn tại đây bằng tiếng Anh hoặc tiếng Việt.

3. **Table S2. Semi-Structured Interview Guide**

Interview questions organized by Consolidated Framework for Implementation Research (CFIR) domain, used to guide semi-structured interviews with study participants about the delivery of genetic services in Vietnam. NA = not applicable.

Q#	CFIR Domain	Interview Question
1	NA	Please briefly tell me what you do and how you interact with genetics in your job.
2	Individuals	Can you tell me what genetic counseling means to you?<br><br>Now, I'm going to read out a short explanation of what genetic counseling is in the US.<br><br>Genetic counseling is a healthcare service that helps people understand how genetic conditions might affect them or their families. A genetic counselor reviews family health history, explains if a condition could be genetic, and provides options for genetic testing. They also help patients understand test results. Their role is to make complex genetic information easier to understand and help patients make decisions.<br><br>In many countries, genetic counseling is a distinct profession. Genetic counselors are trained through two-year Master's programs that focus on both medical genetics and psychosocial counseling.<br><br>In other countries, similar services are often delivered by doctors who receive specialized training. I hope to learn more about how these services are being delivered in Vietnam.
3	Inner Setting + Innovation	Can you walk me through how a patient typically moves through genetic services at your institution? From the first concern that a patient might have something genetic, through to genetic testing, result interpretation, and any follow-up care?<br><br>As you describe this, please tell me who is responsible for each step and what kind of training they have in genetics.<br><br>Follow up:<br>In other countries, genetic counselors take on additional roles, such as

		reviewing a patient's family history to assess their risk of inherited conditions, helping patients emotionally process results that suggest a serious condition, and helping them make decisions, such as whether to pursue genetic testing or to change treatment or pregnancy plans.<br><br>Based on what you just described, are any of these types of support currently being provided at your institution, and if so, by whom?
4	Outer Setting + Innovation	I'm going to read another short description of how genetic counselors work in the US, and then ask for your opinion on whether any similar needs exist for genetic counselors in Vietnam.<br><br>In other countries, genetic counselors work together with doctors in cancer, pregnancy, and pediatric care. In cancer clinics, they help patients understand if their cancer might be inherited and what that means for their prevention, treatment, and their family. During pregnancy, they explain tests that check if the baby may have a genetic condition like Down syndrome. They also support carrier screening, which checks if parents carry genes for inherited conditions that could affect their child. And in pediatrics, they support families in finding answers and planning care when a child has a rare or undiagnosed condition, among other things.<br><br>From your perspective, what kinds of patients or clinical situations would benefit the most from having access to a genetic counselor in Vietnam?
5	Outer Setting + Individuals	What cultural or societal factors might influence the acceptance or rejection of the genetic counseling profession in Vietnam?
6	Inner Setting	Healthcare Providers/Leaders: What challenges do you think might arise if your institution tried to introduce a genetic counselor?<br><br>Geneticists: What challenges do you think might arise if a genetic counselor were introduced into the clinics where you typically collaborate or provide consultations?<br><br>Laboratory representatives: What challenges do you think might arise if a genetic counselor were integrated into your lab's workflow or in the process of returning test results to clinicians and patients?
7	Individuals	What are your thoughts about the idea of establishing genetic counseling as a distinct profession in Vietnam?

8	Closing remark + snowball referrals		Are there any additional thoughts or concerns you have about implementing the profession of genetic counselors in Vietnam?<br><br>Are there others who you believe would offer important perspectives on integrating the profession of genetic counselors in Vietnam, perhaps in different departments, hospitals, or leadership roles?
